# The Impact of Gastric Juice pH on the Intraluminal Therapy for *Helicobacter pylori* Infection

**DOI:** 10.3390/jcm9061852

**Published:** 2020-06-14

**Authors:** Yu-Chio Wang, Yen-Po Chen, Cheng-Yu Ho, Ting-Wen Liu, Cheng-Hsin Chu, Horng-Yuan Wang, Tai-Cherng Liou

**Affiliations:** 1 Department of General Medicine, MacKay Memorial Hospital, Taipei 10449, Taiwan; yuchiowang@gmail.com (Y.-C.W.); Carlos60531@gmail.com (T.-W.L.); mmh4071@gmail.com (C.-H.C.); 2 Department of Medicine, MacKay Medical College, New Taipei City 25245, Taiwan; drchen87@gmail.com (Y.-P.C.); chanyo123@gmail.com (C.-Y.H.); mmh4013@gmail.com (H.-Y.W.); 3 Division of Gastroenterology, Department of Internal Medicine, MacKay Memorial Hospital, New Taipei City 25173, Taiwan; 4 Division of Gastroenterology, Department of Internal Medicine, MacKay Memorial Hospital, Taipei 10449, Taiwan

**Keywords:** *Helicobacter pylori*, gastric juice, acidity, endoscopy, therapy

## Abstract

Background: *Helicobacter pylori (H. pylori)* can be topically eradicated in stomach lumen on endoscopic examination. The procedures of intraluminal therapy for *H. pylori* infection (ILTHPI) include the control of intragastric pH, mucolytic irrigation of the gastric mucosal surface, and a single-dose medicament containing antimicrobial agents. Aims: To detect gastric juice pH and evaluate its impact on the success rate of ILTHPI. Methods: We enrolled 324 patients with upper abdominal discomfort for endoscopic examinations. Among them, 13C-urea breath test was positive in 218 patients, where 100 underwent ILTHPI, and negative in 106. All patients had their gastric juice pH detected and set into three ranges, including normal acidity (pH < 4.0), low-level hypoacidity (pH 4.0–5.5), and high-level hypoacidity (pH ≥ 6.0). The impact of gastric juice pH on the success rate of ILTHPI was evaluated. Results: Distribution of pH level showed no significant difference between two groups of *H. pylori*-infected patients (*p* = 0.942). The eradication rate of ILTHPI is significantly lower in patients with gastric juice pH below 4 (*p* < 0.001). Conclusions: Detection of gastric juice pH in ILTHPI is extremely important. Rapid control of stomach pH at or above 4 for patients prior to ILTHPI is strongly recommended. (NCT03124420).

## 1. Introduction

Several strategies have been proposed to increase the eradication rate of *Helicobacter pylori* (*H. pylori*). However, the widespread increasing rate of resistance to current multiple-dose oral antibiotic therapies calls for alternative therapeutic approaches [1,2]. There has so far been no definitive evidence of immediate eradication of *H. pylori* from single-dose oral therapeutic agents due to the special gastric milieu of *H. pylori*. We established a novel intraluminal therapy for *H. pylori* infection (ILTHPI) in our previous study [3]. Our previous results with ILTHPI suggested that *H. pylori* can be eradicated immediately by administering a single-dose regimen while conducting an endoscopic examination.The procedures of ILTHPI include the control of intragastric pH within a certain range, irrigation of the gastric mucosal surface with a mucolytic agent, and direct application of a single-dose medicament containing antimicrobial agents. Compared to traditional systemic therapy with multiple-dose oral antibiotics, this new strategy of intraluminal therapy provides only a single-dose antimicrobial medicament directly to the mucosal surface colonized by the *H. pylori*, thus mimicking a local therapy. Through administration of a single-dose local application of antibiotics, issues commonly associated with the use of multiple-dose oral antibiotics regarding drug absorption, tissue redistribution, systemic side effects, and patient compliance could be reduced. Since ILTHPI mimics local therapy, as compared with the traditional systemic oral antibiotics therapies, the impact of gastric juice pH on the eradication rate of ILTHPI may be different from that of oral antibiotic therapies, and should thus be studied. We aim to establish a simple and rapid method for the detection and classification of intragastric pH in *H. pylori*-infected patients and to evaluate the impact of gastric juice pH on the success rate of ILTHPI.

## 2. Materials and Methods

### 2.1. Patients

**Figure 1:**
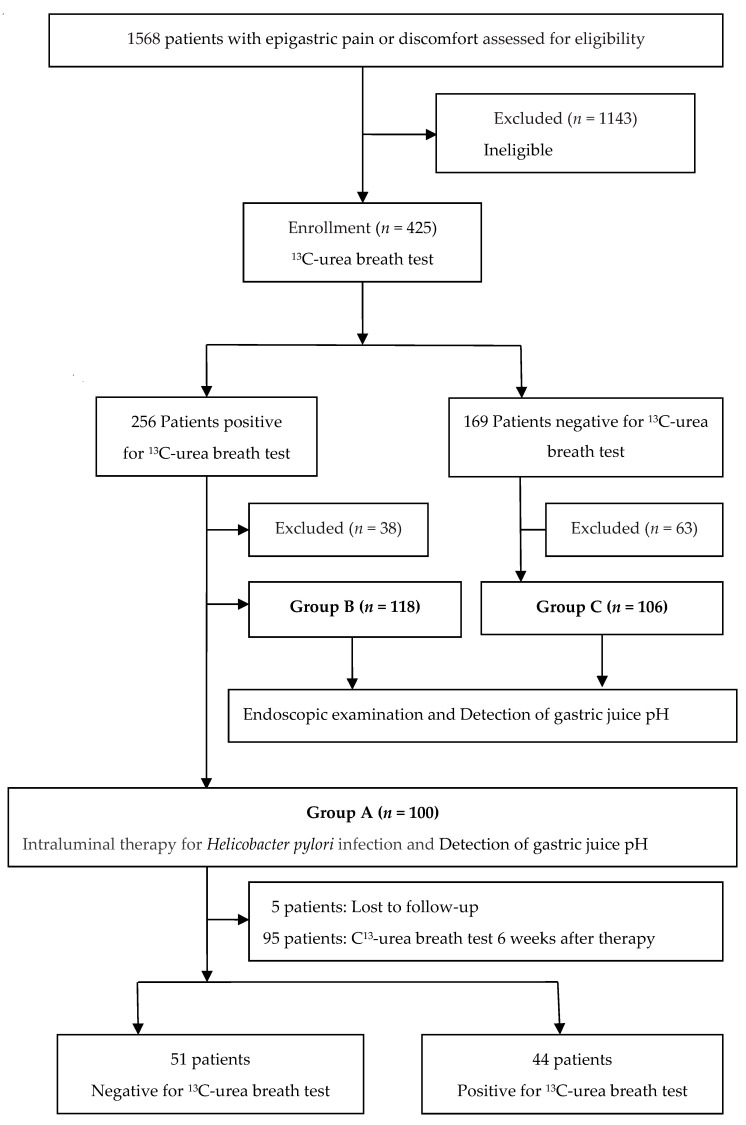
Study flow chart.

This prospective study was conducted at a medical center between April 2017 and April 2019. As detailed in the study flow chart (Figure 1), 1568 consecutive patients with upper abdominal pain or discomfort were assessed for eligibility of ILTHPI, and those eligible were then enrolled to receive ^13^C-urea breath test (^13^C-UBT). Patients with any of the following were excluded from the study: (1) contraindication of endoscopic examination; (2) previous gastric surgery; (3) gastroduodenal deformity, stenosis, or obstruction; (4) gastroduodenal malignancy; (5) previous eradication therapy for *H. pylori*; (6) use of antibiotics or bismuth salts in the previous 4 weeks; (7) use of PPI or H_2_-blocker in the previous 2 weeks; (8) previous allergic history to medications used for the ILTHPI; (9) pregnant or lactating women; (10) severe concurrent diseases (such as advanced renal disease or decompensated liver cirrhosis) or malignancy; and (11) inability or refusal to give written informed consent. We excluded 1143 ineligible patients and performed ^13^C-UBT for the 425 enrolled patients, ages between 20 and 75. ^13^C-UBT was positive in 256 patients and negative in 169 patients. The 1143 ineligible patients, including (1) 132 contraindication of endoscopic examination; (2) 53 previous gastric surgery; (3) 18 gastroduodenal deformity, stenosis, or obstruction; (4) 15 gastroduodenal malignancy; (5) 126 previous eradication therapy for *H. pylori*; (6) 42 use of antibiotics or bismuth salts in the previous 4 weeks; (7) 482 use of PPI or H_2_-blocker in the previous 2 weeks; (8) 37 previous allergic history to medications used for the ILTHPI; (9) 32 pregnant or lactating women; (10) severe concurrent diseases (63 advanced renal disease, 47 decompensated liver cirrhosis, 55 malignancy); and 41 inability or refusal to give written informed consent.

All enrolled patients were invited for endoscopic examination and detection of gastric juice pH. Those tested positive for *H. pylori* infection were also invited to receive concomitant ILTHPI, where 38 declined endoscopic examination, 100 underwent endoscopy with ILTHPI (Group A), and 118 underwent endoscopy without ILTHPI (Group B). From an ethical standpoint, given that our study is a pilot study of ILTHPI, and that the eradication rate of ILTHPI was unknown upon enrollment, we categorized group A and group B based on patient’s willingness, rather than randomization.

For the 169 non-*H. pylori* infected patients, 63 declined endoscopic examination and 106 patients underwent endoscopic examination and detection of gastric juice pH (Group C). All participants provided written informed consent, and the study was approved by the Institutional Review Board of our hospital on 11 April 2017 (IRB number: 17MMHIS020).

### 2.2. Methods

Clinical characteristics of enrolled patients and endoscopic findings were recorded. The procedure of ILTHPI in Group A was performed by a single, experienced senior gastroenterologist, and was strictly followed as detailed in our previous report [3]. The medicament of ILTHPI contains amoxicillin, clarithromycin, and metronidazole. For all participants, a high-resolution electronic endoscope from Olympus Co. (Tokyo, Japan, GIF 260 or 290 series) was used for detection and interpretation mucosal changes, such as ulceration or hyperemia. To avoid contamination affecting the accuracy of pH measurements, premedication (such as mucolytic or defoaming agents) was not administered, except for local pharyngeal anesthesia with 10% lidocaine pump spray. Immediately after the endoscope was inserted into the stomach, 3 to 5 mL of gastric fluid pooled in the stomach was aspirated through a washing pipe (Olympus Co., Tokyo, Japan, EndoTherapy product name: PW-1L-1) inserted into the working channel of the endoscope. The pH of the collected sample was immediately measured using two pH strips (Macherey-Nagel pH-Fix 0.0–6.0 and pH-Fix 4.5–10.0); each scaled in pH 0.5 intervals. Levels of gastric juice pH in each patient are classified into three ranges, including normal acidity (pH <4.0), low-level hypoacidity (pH 4.0 to 5.5) and high-level hypoacidity (pH ≥ 6.0). Ranges of gastric juice pH are compared between Group A and Group B patients with *H. pylori* infection, and are also determined for the 106 subjects without *H. pylori* infection (Group C). The impact of gastric juice pH on the success rate of ILTHPI was evaluated in 100 *H pylori*-infected patients who underwent ILTHPI with medicament containing amoxicillin, clarithromycin, and metronidazole (Group A). The procedures of ILTHPI was detailed in our previous report [3], as follows: Patients were administered two tablets of orally disintegrating lansoprazole (30 mg per tablet) before ILTHPI and another two tablets 8 to 10 h after ILTHPI. During ILTHPI, an endoscopic apparatus and washing pipe from Olympus Co. (Tokyo, Japan, EndoTherapy product name: PW-1L-1) were used to irrigate the gastric mucus with acetylcysteine (12 mg/mL) solution to remove the mucus on the gastric mucosa. The total dosage of acetylcysteine was less than 140 mg/kg. Three types of antibiotic powders (3 g of amoxicillin powder for suspension, 2 g of crushed metronidazole tablets, and 1 g of crushed enteric-coated clarithromycin tablets in powder form) were mixed with 60 mL (6 g) of sucralfate suspension and 120 mL of distilled water and were applied to all surfaces of the gastric mucosa and the duodenal mucosa of the duodenal bulb as evenly as possible using the same washing pipe. During the endoscopic examination and ILTHPI, each patient was sedated with intravenous midazolam (5 mg). After ILTHPI, patients were asked to rest for 30 min before leaving for the effects of sedation to wear off. However, they were allowed to take meals if they did not experience abdominal discomfort. The^13^C-UBT was used to assess the successful eradication of *H. pylori* 6 weeks after ILTHPI. The eradication rate of ILTHPI was calculated for per-protocol patients, and those who failed to return for follow-up ^13^C-UBT were excluded.

### 2.3. Statistical Analysis

Unless otherwise indicated, values were expressed as mean± standard deviation (SD). Categorical data were compared using the *χ^2^* test or Fisher’s exact test, as appropriate. The demographic data were expressed as mean ± SD. Student’s *t*-test was used to compare the mean values of continuous variables. All reported p-values were based on two-sided tests and considered statistically significant if less than 0.05. All data were analyzed using SPSS version 21.0 (IBM, Armonk, NY, USA).

## 3. Results

### 3.1. Patient Characteristics

Table 1 shows the clinical characteristics of all three groups of patients. There are no significant differences in any of thedemographic characteristics among the three groups, except that the BMI is significantly higher in *H. Pylori*-infected patients (*p* = 0.019 for Group A vs. Group C and *p* = 0.020 for Group B vs. Group C). There is no significant difference in BMI between Group A and Group B *H. Pylori*-infected patients (*p* = 0.864).

Table 2 shows the endoscopic characteristics of all three groups of patients. There is no significant difference in endoscopic findings among the three groups regarding proportions of peptic ulcer disease, gastritis, or normal appearance. However, sub classification of endoscopic gastritis revealed that gastritis in cardia is significantly higher in proportion in *H. pylori*-infected patients (*p* = 0.001 for Group A vs. Group C and *p* = 0.002 for Group B vs. Group C), while gastritis in antrum is significantly higher in non-infected patients (*p* < 0.001 for Group A vs. Group C and *p* < 0.001 for Group B vs. Group C). Gastritis in corpus is also significantly higher in non-infected patients (*p* = 0.017 for Group A vs. Group C and *p* = 0.001 for Group B vs. Group C). There is no significant difference between Group A and Group B *H. pylori*-infected patients regarding gastritis in either cardia (*p* = 0.786), corpus (*p* = 0.772), or antrum (*p* = 0.782).

### 3.2. Validation of the pH Strip

As shown in Table 3, levels of gastric juice pH are set into three ranges for all patients. There is no significant difference in the distribution of pH level between *H. pylori*-infected Group A and Group B patients (*p* = 0.942). Levels of pH in all non-infected patients (Group C) are at 3.5 or less (range: 1.0–3.5/mean ± SD: 1.52 ± 0.88), which is comparable with the range of gastric juice pH (1.5–3.5) in the lumen of a normal human stomach [4].

### 3.3. The Impact of Gastric Juice pH on the Success Rate of ILTHPI

Table 4 shows the impact of gastric juice pH on the success rate of ILTHPI in 100 *H. pylori*-infected patients who underwent ILTHPI with medicament containing amoxicillin, clarithromycin, and metronidazole (Group A). The eradication rates of ILTHPI were 33.3% (15/45, 95% confidence interval (CI):21.3% to 48.0%), 71.4% (5/7, 95% CI: 35.2% to 92.4%), and 72.1% (31/43, 95% CI: 57.2% to 83.4%) for patients with ranges of gastric juice at pH ≤ 3.5, 4–5.5, and ≥6, respectively. The eradication rate of ILTHPI is significantly lower in patients with gastric juice pH ≤ 3.5 than in patients with gastric juice pH at or above 4 (*p* < 0.001; α = 0.05, power = 0.975). There is no significant difference in the eradication rate between patients with gastric juice pH level ≥ 6 and those with gastric juice pH between 4 and 5.5 (72.1% vs. 71.4%; *p* = 1.000). Although the eradication rate of ILTHPI is higher in patients with gastric juice pH between 4 and 5.5 than in patients with gastric juice gastric juice pH ≤ 3.5 (71.4% vs. 33.3%), there is no significant difference statistically (*p* = 0.092; α = 0.05, power = 0.486) due to small sample size. To show a statistically significant difference in eradication rate between an average of 71.4% and 33.3, a sample size of 26 patients for each group is estimated (α = 0.05, power = 0.80).

## 4. Discussion

Geographic differences in health insurance systems, prevalence of gastric cancer, and the availability of medications and endoscopy contribute to variances in the indications for *H. pylori* eradication and endoscopy. Over 99% of the population in Taiwan is under the National Health Insurance (NHI) system sponsored by the government. The NHI program provides full coverage for medications used to eradicate *H. pylori* and endoscopy for pretreatment screening and post-treatment surveillance given the high prevalence of *H. pylori* infection and gastric cancer in Taiwan [3]. The cost of endoscopic examination is rather affordable, at 2000 New Taiwan dollar (equivalent to 60 United States dollar), in Taiwan and is entirely covered by our NHI for patients with upper abdominal pain or discomfort regardless of *H. pylori* infection. Additionally, ILTHPI achieves concomitant eradication of *H. pylori* with single-dose medicaments on endoscopic examination, which eliminates the need for subsequent multiple-dose oral antibiotic therapies, which is of grave importance in view of the heavy expenses *H. pylori* eradication incurs in health systems.

There are limitations in our study. First, as a pilot study in ILTHPI, and also with the aim of detecting the gastric juice pH, most of the excluded patients in our study are actually suitable for the procedures of ILTHPI, including the 126 previous eradication therapy for *H. pylori*, the 482 use of PPI or H_2_-blocker in the previous 2 weeks, and the 42 use of antibiotics or bismuth salts in the previous 4 weeks. Second, we enrolled patients in group B who declined ILTHPI but agreed to receive endoscopic examination and detection of gastric pH only to validate the reliability of pH strip in distinguishing the three pH ranges. However, it would certainly be better to include a comparison arm received regular oral antibiotics treatment and measured pH in our future studies.

The relationship between *H. pylori* infection and obesity or overweight is controversial. A meta-analysis study from China demonstrated the relevance between obesity and *H. pylori* infection [5]. Our study also revealed that the BMI is significantly higher in *H. pylori*-infected patients (Group A and Group B) than those without *H. pylori* infection (Group C) (Table 1). There is no significant difference in BMI between Group A and Group B patients (*p*= 0.864). Although no parameters showed a significant difference among group A, group B, and group C, each parameter was higher in percentage in group C than in the other two groups with respect to non-steroid anti-inflammatory drug, smoking, and alcohol consumption, which are risk factors associated with development of gastritis and peptic ulcers, as shown in Table 1, and may thus be the cause of a high rate of gastritis and peptic ulcer in non-*H. pylori*-infected patients (Table 2).The occurrence of gastric carditis is significantly higher in *H pylori*-infected patients (Group A and Group B) than non-infected patients (Group C) (Table 2). Persistent *H. pylori* colonization resulting in subsequent chronic inflammation of cardia has been established in many studies. The rate of colonization detected by rapid urease test in gastric cardia was 90% in *H. pylori*-infected patients [6], and was also significantly higher in patients with cardiac cancer (81.5%) and gastric carditis (80.1%) [7].

Both the degree and the duration of acid suppression serve as major contributing factors for higher rate of *H. pylori* eradication by oral antibiotics [8]. Many studies have suggested that maintenance of intragastric pH at ideally 4 or above stabilizes the pharmacological properties of the administered antibiotics [8,9,10], and that sustained control of intragastric pH at 6 or above stimulates the replication of *H. pylori* and increases the bactericidal efficacy of oral antibiotics [8,10,11,12]. However, as a single-dose local application of antibiotics to the gastric mucosal surface the effects ofgastric juice pHon the eradication rate of ILTHPI may be different. Given the experience from traditional oral antibiotic treatment, and that the pH strips applied in this study are scaled at 0.5 intervals, the levels of gastric juice pH are set into three ranges, including normal acidity (pH < 4.0), low-level hypoacidity (pH 4.0 to 5.5), and high-level hypoacidity (pH ≥ 6.0). Our study demonstrated that pH strip detection is a reliable approach for the evaluation and classification of gastric juice pH (Table 3). The eradication rate of ILTHPI is significantly lower in patients with normal acidity (pH < 4.0). However, there is no significant difference between patients with low-level hypoacidity (pH 4.0 to 5.5) and high-level hypoacidity (pH ≥ 6) (Table 4).

Several factors may contribute to the lower eradication rate of ILTHPI with medicament containing amoxicillin, clarithromycin, and metronidazole. Amoxicillin, clarithromycin, and metronidazole are stable in aqueous solutions at pH levels ranging from 4.0 to 7.0, 5.0 to 8.0, and 2.0 to 7.0, respectively. In gastric juice samples at pH level of 2.0, the degradation half-lives of these antibiotics are 15.2 ± 0.3 h, 1.0 ± 0.04 h, and ~800 h, respectively. Half-lives of the aforementioned drugs in the gastric juice samples at pH level of 7.0 are all >68 h. Clarithromycin degrades rapidly under normal gastric pH level. However, amoxicillin and metronidazole are sufficiently stable under the same conditions [9]. The influence of pH on the antimicrobial susceptibility of *H. pylori* was also reported in previous studies. Ampicillin is bactericidal at pH levels of 4.5 and 7.4, but not at a pH level of 3.0, since *H. pylori* decreases the expression of its cell envelope and division genes, and thus loses the ability of cell division under such levelsof acidity [10]. Therefore, amoxicillin and clarithromycin are less effective at pH levelsless than 4, leading to a negative impact onsuccess rate in patients with normal acidity of gastric juice. On the other hand, metronidazole is relatively stable in an acidic environment (pH 2.0–7.0). With ~800-h degradation half-life in gastric juice samples at a pH level of 2.0 [9], lower pH did not alter the bactericidal effect of metronidazole. We used a metronidazole-containing medicament for ILTHPI in our study, which may play an important role in achieving eradication of *H. pylori* for patients with gastric juice pH of less than 4.

We did not find a significant difference in the eradication rate of ILTHPI between patients with low-level hypoacidity (pH 4.0 to 5.5) and high-level hypoacidity (pH ≥ 6.0). When the acidity of the environment increased from pH 6.0 to pH 5.0, the 90% minimum inhibitory concentration (MIC_90_) for levofloxacin increased significantly across break points. However, the MIC_90_ for amoxicillin, clarithromycin, tetracycline, moxifloxacin, and gemifloxacin and the MIC_50_ for metronidazole only showed mild elevations [11]. Additionally, direct application of a single-dose antibiotic on the gastric mucosal surface was able to achieve an extremely high level of antibiotic dosage compared with the traditional systemic delivery of multiple-dose oral antibiotic. The immediate eradication of *H. pylori* via ILTHPI also indicates that local application of medicaments containing metronidazole and clarithromycin could enhance the bactericidal effect of amoxicillin to a less replication-dependent level. Since ILTHPI provides immediate eradication of *H. pylori* through local application of high-dose antibiotics, there may be less impact of antibiotic MIC and the replication of *H. pylori* on the eradication rate as compared with the traditional approach.

Aside from the control of intragastric pH level, the procedures of ILTHPI also include irrigation of the gastric mucosal surface with a mucolytic agent, and application of a single-dose medicament containing antimicrobial agents to the mucosal surface of stomach. Approximately 80% of *H. pylori* reside in the mucus layer of the human stomach [13], while the remaining colonizes reside on the underlying mucosal epithelial cells, and only a very small percentage of *H. pylori* survive in the gastric juice. The stomach consists of two layers of mucus: the firmly adherent inner mucus layer and the loosely adherent outer mucus layer [14,15]. The inner layer is firmly attached to the gastric mucosa and is difficult to remove, while the outer layer is less dense and can be easily removed by irrigation and suction. After irrigation of the mucosal surface with a mucolytic agent, the gastric juice and the detached outer mucus layer are removed via suction, while the firmly adherent inner mucus layer may remain attached to the epithelial surface. Gastric mucin, a high molecular weight glycoprotein, is responsible for providing the gel-forming property of the gastric mucus, and serves a protective function at a pH level of less than 4 [16,17]. The conformational change of mucin facilitates cross-links among mucin macromolecules through hydrophobic interactions at pH level of less than 4, which in turn leads to a solution-to-gel transition (sol–gel) [16]. Bulk rheology measurements in the linear viscoelastic regime also demonstrated that gastric mucin undergoes a pH-dependent sol–gel transition from a viscoelastic solution at neutral pH to a viscoelastic gel under acidic conditions, with the transition occurring near pH 4 [17].

When exposing gastric mucus to pH levels below 4, the gel-forming mucus layer serves as a critical barrier for blocking the diffusion of antibiotic particles applied from the gastric lumen, and also plays a role in ensuring the colonization of *H. pylori* in the adherent mucus gel and its underlying epithelial layer [18]. Gastric mucus undergoes a reversible pH-dependent transition between a soft gel and a viscous polymer. The glycoprotein macromolecules of mucin transform into solution phase under neutral pH, and change to the gel phase under low pH (<4) [17]. The control of stomach acidity to a level of hypoacidity prior to ILTHPI leads to a gel–sol transition of the mucus layer, and is favorable for both the irrigation of gastric mucus and the application of antimicrobial agents. In addition, higher level of gastric juice pH, at or above 4, inactivates the activity of pepsin [19] and brings about an increased content and enhanced immunocompetence of anti-*H. pylori* immunoglobulin A, which also contributes to higher *H. pylori* eradication rate [20].

As the pH level of the gastric lumen becomes more acidic, even mucus is gelled slightly stronger at pH 2 than at pH 4; *H. pylori*, however, still manages to achieve penetration by altering the rheological properties of the mucus via increased urease activity [21,22]. The *H. pylori* UreI channel favors urea passage at low pH [23] and creates a microenvironment by hydrolyzing urea present in the stomach to ammonia and carbon dioxide, thus causing widespread pH elevation. Elevation of pH level liquefies the mucus in the vicinity of each bacterium [22]. In addition, bicarbonate secretion creates and maintains a gradient from pH 1–2 in the gastric lumen to approximately neutral pH at the epithelial surface [24]. *H. pylori* has been shown to exhibit a pH tactic response toward elevated pH level [25,26], and may be able to swim faster at low pH, since its flagellar motor is powered by a proton motive force [27]. Thus, *H. pylori* is able to promptly evade the highly acidic lumen, swim through the mucus layer towards the epithelial surface, and away from the more acidic outer mucous layer. A higher proportion of *H. pylori* are distributed inthe deeper layer of attached mucous than in the unattached outer layer, which might be unfavorable for ILTHPI.

Gastric pH is normally kept below 4.0 [28]. Pepsin is most active around a pH level of 1.5 to 2, assuming its inactive form being at pH levels above 4 [19]. To prevent mucosal injury from acid and pepsin during the window between the irrigation of the mucosal surface and the renewal of the mucus layer, and to increase the eradication rate, we tried to control the intragastric pH to a level above 4 on the day of the ILTHPI through the administration of high-dose lansoprazole [29] (by orally disintegrating lansoprazole 60 mg immediately before ILTHPI and another 60 mg 8 to 10 h after). Although chronic *H. pylori* infection could result in the reduction of acid secretion to a level of hypoacidity from atrophy of the oxyntic mucosa [30], about 46% (46/100) in Group A and 46.6% (55/118) in Group B *H. pylori*-infected patients hadlevels of gastric juice pH that were still less than 4. A more effective strategy for rapid control of intragastric pH prior to ILTHPI is of paramount importance when it comes to improving the eradication rate of *H. pylori* for medicaments containing amoxicillin and clarithromycin. Whether a more rigorous control of stomach pH at or above 6 prior to ILTHPI for medicaments containing other antimicrobial agents would increase the success rate of ILTHPI requires further study. Although Vonoprazan [31] (a potassium-competitive acid blocker) achieves a more rapid and sustained control of intragastric pH level at 6 or above, it is currently not available in many countries.

Aside from the control of intragastric pH level, several factors may influence the eradication rate of ILTHPI. The antibiotics resistance, high bacterial load, the use of certain mucolytic agents and novel antimicrobial agents, the administered dosage and formulation of medicaments, and the specified devices for the irrigation and application of medicaments might affect the efficacy of ILTHPI. However, since our study is a pilot study of ILTHPI, the aforementioned factors require further investigation for confirmation.

Although the overall *H. pylori* eradication rate of ILTHPI was 53.7% (51/95) in our study, the eradication rate markedly improved from 33.3% (15/45) to 71.4% (5/7) or 72.1% (31/43) when the gastric juice pH was maintained at or above 4 (Table 4). In addition, the development of specialized devices, suitable mucolytic agents, and novel antimicrobial agents [32] for ILTHPI might further increasethe rate of eradication to above 80%. However, before any improvement in the above aspects canbe achieved, we strongly suggest the detection of gastric juice pH in ILTHPI and a more rapid control of stomach pH at the level of 4 or above prior to ILTHPI. To overcome a gastric juice pH below 4, we suggest the use of a number of days of PPI or P-CAB to control stomach pH above or at 4 before ILTHPI in clinical practice. For patients whose gastric juice pH is still detected below 4 during the ILTHPI, we recommend either the choice of a medicament containing acid-stable antibiotics (such as metronidazole), or the use of a novel acid-stable antimicrobial agentfor ILTHPI, if available in the future. However, the use of high-dose PPI or P-CAB for a number of days before traditional multiple-dose oral antibiotics therapy could also be considered before the development of suitable acid-stable novel agents for ILTHPI.

Although the endoscopy costs are relatively low in our country, the approach of endoscopy with ILTHPI may not be cost-effective in other locations in which the cost of endoscopy and associated sedation are high. Regardless, for *H. pylori* infected patients, when the indications of endoscopy come, the concomitant ILTHPI could provide an opportunity to eradicate *H. pylori* immediately, especially for patients whose gastric juice pH is detected at or above 4. Since the cost of a pH strip is quite affordable at 8 NTD/2 strips (0.27 USD/2 strips), and can be read off within 3 s, it should be an ideal and appropriate method for the detection of gastric juice pH. Mean while, the level of gastric juice pH could assist further decision making with respect to the choice of suitable medicaments for ILTHPI, or it might be helpful in the selection of therapeutic strategies for traditional oral antibiotics treatment. In addition, the average duration of ILTHPI was only 11 min and 4 s. in our study, inventing suitable devices specifically tailored to the ILTHPI will certainly shorten the duration of the ILTHPI, and hence make it exempt from sedation. The specified devices may include a more effective pumping machine, a more powerful shower nozzle for irrigation, and a specifically designed sprayer nozzle for the application of medicaments [3].

## 5. Conclusions

Detection of gastric juice pH in ILTHPI is extremely important. A more effective strategy for the rapid control of intragastric pH level at or above 4 for patients prior to ILTHPI is strongly recommended.

## Figures and Tables

**Table 1:** Clinical characteristics of patients at study entry.

Characteristics	Group A ^†^ (*n* = 100)	Group B ^†^ (*n* = 118)	Group C ^‡^ (*n* = 106)
Age (years, mean ± SD /range) *	52.1 ± 10.3 (24–74)	51.8 ± 11.6 (26–73)	50.7 ± 11.2 (23–74)
Gender (M/F) *	47/53	57/61	56/50
NSAID ingestion *	21 (21.0%)	26 (22.0%)	35 (33.0%)
Smoking *	17 (17.0%)	24 (20.3%)	28 (26.4%)
Alcohol consumption *	7 (7.0%)	10 (8.5%)	16 (15.1%)
Ingestion of tea *	30 (30.0%)	40 (33.9%)	42 (39.6%)
Ingestion of coffee *	39 (39.0%)	45 (38.1%)	49 (46.2%)
BMI (kg/m^2^, mean ± SD/range) **	25.9 ± 4.4 (17.5–36.5)	25.8 ± 4.2 (17.6–37.4)	24.5 ± 4.1 (17.3–36.1)

^†^*Helicobacter pylori* infected patients ^‡^ non-*H pylori* infected patients SD: standard deviation. NSAID: non-steroid anti-inflammatory drug. BMI: Body Mass Index. * *p* > 0.05 among Group A, Group B and Group C. ** *p* = 0.864 for Group A vs. Group B; *p* = 0.019 for Group A vs. Group C, and *p* = 0.020 for Group B vs. Group C.

**Table 2:** Endoscopic findings of patients at study entry.

Endoscopic Findings	Group A ^†^ (*n* = 100)	Group B ^†^ (*n* = 118)	Group C ^‡^ (*n* = 106)
Normal *	15	16	9
Gastritis *	85	102	97
(antrum **/corpus **/cardia ***)	(39/66/57)	(49/71/65)	(84/86/36)
Peptic ulcer disease *	28	32	38

^†^*Helicobacter pylori* infected patients. ^‡^ non-*H pylori* infected patients. * *p* > 0.05 for proportions of peptic ulcer disease, gastritis or normal appearance among Group A, Group B and Group C. ** Group A and Group B have lower rate of endoscopic findings of antrum and corpus inflammation than Group C (*p* < 0.05). *** Group A and Group B have higher rate of endoscopic findings of carditis than Group C (*p* < 0.05).

**Table 3:** Distribution in the ranges of gastric juice pH level.

Gastric Juice pH	Group A ^†^ (*n* = 100) *	Group B ^†^ (*n* = 118) *	Group C ^‡^ (*n* = 106)
≤3.5	46	55	106 ^§^
pH 4–5.5	9	12	0
pH ≥ 6	45	51	0

^†^*Helicobacter pylori* infected patients ^‡^ non-*H pylori* infected patients. * *p* = 0.942 for distribution in the ranges of gastric juice pH between Group A and Group B. ^§^ Mean ± SD (standard deviation) is 1.52 ± 0.88 (range: 1.0–3.5) for 106 Group C patients.

**Table 4:** The impact of gastric juice pH on the success rate ofintraluminal therapy for *Helicobacter pylori* infection (*n* = 100).

Range of Gastric Juice pH	Patients Number (Lost to Follow Up)	Eradication Rate
pH ≤ 3.5 *	46 (1)	15/45 (33.3%) **
pH 4–5.5 ***	9 (2)	5/7 (71.4%) **
pH ≥ 6 ***	45 (2)	31/43 (72.1%)

* *p*< 0.001 for the eradication rate comparing patients with gastric juice pH ≤ 3.5 vs. pH ≥ 4 (α = 0.05, power = 0.975). ** *p* = 0.092 for patients with gastric juice pH 4–5.5 vs. ≤3.5 (α = 0.05, power = 0.486). *** *p* = 1.000 for the eradication rate comparing patients with gastric juice pH ≥ 6 vs. 4–5.5.
